# Tissue Engineering Using Human Mineralized Bone Xenograft and Bone Marrow Mesenchymal Stem Cells Allograft in Healing of Tibial Fracture of Experimental Rabbit Model

**Published:** 2012-02-01

**Authors:** J Ai, S Ebrahimi, A Khoshzaban, T S Jafarzadeh Kashi, D Mehrabani

**Affiliations:** 1 Department of Tissue Engineering and Cell Therapy, School of Advanced Medical Technologies, Tehran University of Medical Sciences, Tehran, Iran; 2 Research Center for Science and Technology in Medicine, Tehran University of Medical Sciences, Tehran, Iran; 3 Stem Cell and Transgenic Technology Research Center, Department of Pathology, Shiraz University of Medical Sciences, Shiraz, Iran; 4 Department of Biology, Faculty of Sciences, University of Tarbiat Moallem, Tehran, Iran; 5 Research Center and Iranian Bank of graft products, Tehran, Iran; 6 Department of Dental Materials, Faculty of Dentistry, Tehran University of Medical Sciences, Tehran, Iran

**Keywords:** Bone, Xenograft, Allograft, Mesenchymal stem cells, Healing, Rabbit

## Abstract

**Background:**

Bone healing and its reconstruction in fractures, especially in long bones are of particular importance in regenerative medicine. This study compares the bone healing rate after a human xenograft of mineralized bone and together with an allograft of bone marrow mesenchymal stem cells (MSCs) in an experimental tibial bone fracture rabbit model.

**Methods:**

In fall 2009, twenty New Zealand white rabbits were randomly divided into 2 equal groups. In both groups, a 5 mm segmental defect was created in the right tibia. In group A, a scaffold pin was seeded with allogenic rabbit MSCs and was placed in the defect area and in group B, the defect was filled with an unseeded pin human mineralized bone xenograft. An untreated defect was induced in the left tibia of all animals serving as the control. After 4-8 weeks, the segmental defects were histologically evaluated and also by a compressive test.

**Results:**

In groups A and B, healing and formation of new bony tissue were significantly more than the control group and with a significant less inflammation.

**Conclusion:**

Tissue engineering of mineralized bone xenograft and MSCs allograft may be significant steps in bone healing and regenerative medicine.

## Introduction

Bone as part of skeletal system is responsible for mechanical support, body shape and movement. In addition, its role in mineral homeostasis and in energy metabolism was previously shown.[1] An autologous bone graft is used in patients with large bone defects but still is not easily available for about 40% of subjects.[2] Healing in large bone defects using adult stem cells together with ceramic scaffolds is a today need; but the biological mechanisms in this relation are not still completely clear.[3] In case of damage in bone integrity (e.g. after fracture), mesenchymal stem cells (MSCs) play an important role in tissue engineering. MSCs are multipotent cells of mesodermal origin capable of differentiating into osteoblasts, chondrocytes, adipocytes, tenocytes and myoblasts.[4][5] Apart from bone marrow, they are found in endosteum of the trabecular bone, and the periosteum too.[6] Some of cytokines released from the damaged bone matrix and from degranulated thrombocytes result into formation of a mixture of biologically active protein that can affect MSCs chemotactically. MSCs derived from periosteum and bone marrow when transferred to the site of bone damage, they continue to multiply and differentiate into osteoblastic, chondroblastic and fibroblastic cell lines[7] and participate in production of bone and cartilage tissues that form a callus at the fracture site.[8][9]

Fixation of the fracture site has an important role in repair of bony tissue. The under-load bone fragments may experience certain motions by unknown mechanisms affecting the morphologic features of repair in the fracture site.[10] Intra-medullary pinning has been previously used in fixation of bony tissue in small animals.[11] MSCs are the most used cell types in tissue engineering of bony tissue, both in vivo and in vitro.[3] These cells can be easily isolated from bone marrow and expanded through several passages while retaining their multipotent differentiation capacity. Lowimmunogenicity of MSCs both in vitro and in vivo, suggest their utility for autologous as well as allogeneic transplantations. Besides their differentiation capabilities, MSCs take part in matrix remodeling and possess paracrine activities particularly in the microenvironment of a wound.[2] Therefore, in use of MSCs, a number of osteoinductive carriers have been tested based on synthetic polymers, DBM, hydrogel, titanic fibers, natural coral and synthetic bioceramics based on hydroxyapatite and tricalcium phosphate.[12][13][14]

In this study, the bone healing rate after a tissue engineering technique of bone xenograft scaffolds from human bone is compared with an allograft of bone marrow derived MSCs in an experimental tibial fracture rabbit model.

## Materials and Methods

In fall 2009, twenty 6 months old male New Zealand white rabbits weighing 2.5 kg provided from Laboratory Animal Center of Shiraz University of Medical Sciences were divided into 2 equal groups. In both groups, a 5 mm segmental defect was induced in the right tibia. To compare the healing rate of the defect in the right tibia, in group A, a scaffold pin was seeded with allogenic rabbit MSCs and was implanted in the defective area. In group B, the defect was filled with just a human mineralized bone xenograft (unseeded pin). For control group, in the left tibia of all animals, an identical defect was induced without any treatment.

Animal selection, all experiments, subsequent care and the sacrifice procedures were all adhered to the guidelines and were under the supervision of the Animal Care Committee of Iran Veterinary Organization. The study was approved by the Ethics Committee of Tehran University of Medical Sciences. The animals were kept individually in one cage in an ambient temperature of 21±2°C and a 65-70% relative humidity. They were fed with a balanced diet and had free access to water and had a free mobilization condition.

The scaffold was a human bone fragment with a size of 1x1x30 mm provided from human femoral bone with a compact and cortical surface and parallel grooves that were used for cell seeding. These fragments were sterilized by C0-60 γ-rays before use. The scaffold pin was seeded with cells cultured for 3 days in the media.

A compressive test with a rate of 1.0 mm/min was applied for the bone scaffolds (Santam STM-20) and the load and displacement data were recorded in the computer to provide the stress-strain curves and to determine the compressive strength and stiffness of the scaffolds. The breaking load was obtained from the slope of the curve. Bending modulus (e) and strength (δ) were determined using δ=3FL/2bd(2) and e=L3F/4wh(3)d formulas.

Under general anesthesia, the bone marrow was derived from the iliac crest using a 3 mm in diameter of sterile bone marrow aspiration needle. After touching the marrow space and removing the trocar, bone marrow was aspirated using a 2 ml syringe containing 0.1 ml heparin. The aspirated marrow was mixed in a 15 ml falcon tube (Nunc, Germany) containing 5 ml of DMEM (Gibco, USA) and 4 mm ficol (General, Netherland). The marrow aspirate was then homogenized with a pipette, then plated in a T75 culture flask (Nunc, Germany) containing DMEM (Gibco, USA) and 10% FBS (Bio-sera, UK) after they were centrifuged at 2000 rpm for 20 min (Hettich Universal 320 R , Germany). After 24 h, nonadhesive cells (hema-topoietic cells) were eliminated by medium change and the adhesive cells were washed once with PBS (Gibco, USA) and incubated at 37°C and 5% CO2. The medium was changed twice a week. Adherent spindle-shaped cells were obtained from the primary culture after 3 passages. At 80-90% confluence, cells were harvested with trypsin/EDTA (Bio-Sera, UK) and replated by splitting, usually 1:3 at a density of 50–60%. Cells of 3rd passage were analyzed by flowcytometry using MSCs with typical cell surface markers including CD90, CD130, CD34 and CD45 (Stem cell, UK).

Bone scaffolds (pin) were preserved for 2 h in DMEM medium. 1x106 cells from the 3rd passage were resuspended in the medium. The cell suspension was added to the pin using a 26-gauge needle to inject 1 ml until the scaffold material was soaked completely in the suspension. The pin was then placed in an incubator at 37°C for 4 h. Subsequently, the respective media were added and the pin was incubated at 37°C in 5% CO2. They were rinsed twice with PBS, fixed in 2.5% glutaraldehyde in 0.1 M phosphate buffer for 2 h and prepared for scanning electron microscopy (SEM) (Philips XL30, Japan) to determine the cell adhesion and penetration. The scaffold pin was implanted after 3 days of culture with allogenic rabbit MSCs.

**Fig. 1:**
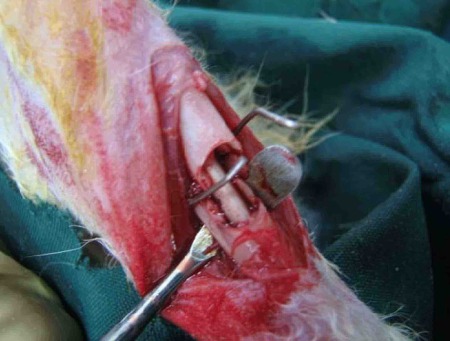
Transplantation of pin seeded with MSCs into segmental tibia defect fixed with ligature wire in rabbit model.

A unilateral 5 mm defect was created in the middle part of the tibia. The rabbits were anesthetized by intramuscular injection of ketamine hydrochloride (50 mg/kg, Unitech, Germany) and xylazine (5 mg/kg, Unitech, Germany). An antibiotic (4 mg/kg, Razi, Iran) was supplied preoperatively. Following a 3 cm supramedial incision over the distal tibia, the soft tissues were dissected and the bone was exposed after gentle retraction of the muscles. A 5 mm segmental tibial defect in the right and left tibias of the rabbits were created under irrigation with 0.9% sterile saline solution. Created defects in the right tibias were used for treatment groups and the left tibias were used as control group. The periosteum was removed with the bone, and 5 ml of periosteum was deprived from each side of the remaining tibia. The gap was irrigated with sterile physiological saline solution and in group A, the pin and MSCs or in group B, the pin only filled the gap. For fixation of these segmental defects in groups A and B and the control group, a ligature wire was applied. Muscles, fascia and skin were separately closed over the defect with 4–0 resorbable sutures (Norderstedt, Germany) (Figure 1). After 4 weeks, five animals and after 8 weeks, 5 more animals were sacrificed from each group and their tibia were removed and fixed in 10% formalin for one week for histological analysis.

Formalin-fixed samples were decalcified in 10% nitric acid for one week, then dehydrated in an ethanol series and embedded in paraffin using standard histological techniques. Five-micrometer serial sections were cut. Sections were stained with hematoxylin and eosin (H and E) to identify any inflammatory response based on infiltration of lymphocytes around the defective site as described by Tanaka et al.[15]

Data analysis was performed with SPSS software (Version 14.0, Chicago, IL, USA). Data were presented as the mean±standard error of the mean (SEM). Statistical comparisons were made by one way ANOVA and when significant differences were observed, Tukey test was employed for multiple comparisons. Statistical significance was considered at p<0.05.

## Results

Formation of new bony and vascular tissues was visible adjacent to the scaffold pin edge 4 weeks postimplantation. Four weeks post-implantation, all animals in group A treated with pin and in group B treated with pin and MSC showed a fully recovered motor response. The implanted scaffolds degraded clearly and were replaced by newly formed bony and cartilage tissues. Many cells, such as chondrocytes and osteocytes were noticed in the implantation site. New bony tissue was present within the marrow cavity. The new bony tissue in the defective region had completely been remodeled.

**Fig. 2:**
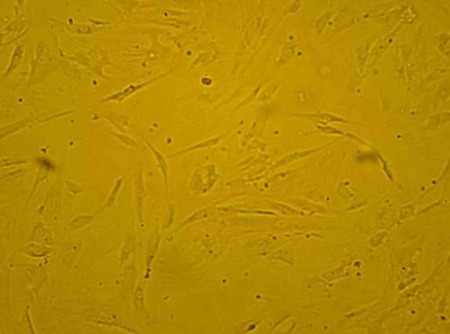
Microscopic view of bone marrow-derived mesenchymal cells in the primary culture (40x).

**Fig. 3:**
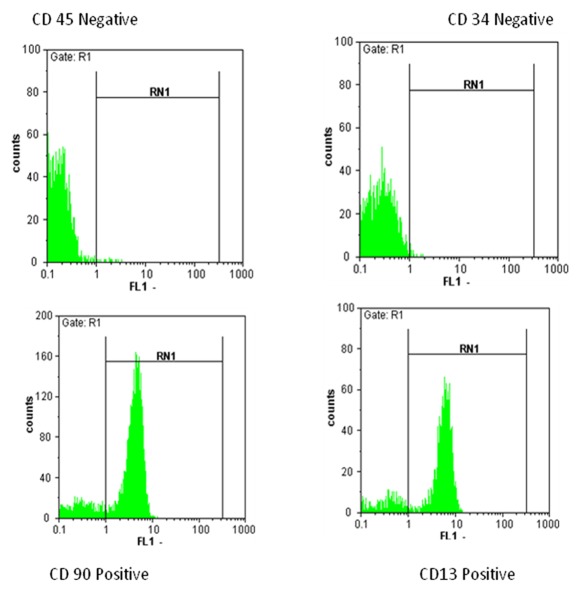
Flow-cytometry analysis of the rabbit bone marrow adherent cells after 3 passages.

**Fig. 4:**
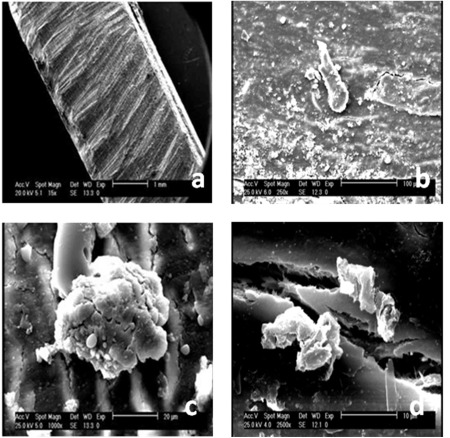
SEM micrograph of pin seeded with MSCs after 3 days of culture in vitro. a) SEM image of the pin surface (bar=1 mm); b) MSCs were seeded on the surface of the pin (bar=100 μm); c) A colony from MSCs on the surface of the pin (bar=20 μm); d) Parallel grooves and colonies from MSCs on the surface of the pin (bar=10 μm).

**Fig. 5:**
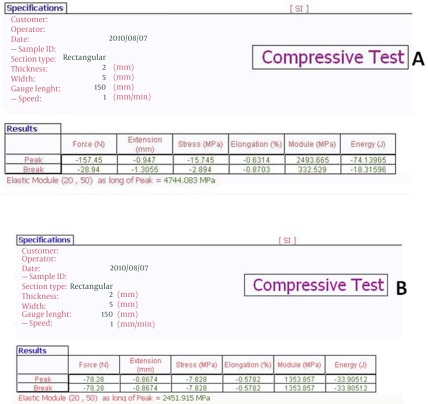
A. The results of compressive test for pin. B. The results of compressive test for pin bending test. Data were automatically recorded and stored in a computer.

MSCs derived from bone marrow had a similar spindle-shaped morphology which were negative for hematopoietic (CD34, CD45) and positive for mesenchymal markers (CD13, CD90). Results for flowcytometry analysis were shown after 3 days of cell seeding and SEM observations showed the presence of MSCs on the scaffold (Figure 2, Figure 3, Figure 4). The compression breaking load for scaffold was 157.5 N; and for compressive modulus and compressive strength were 2493.6 MPa and 15.74 MPa respectively (Figure 5A). The compression breaking load for scaffold bending test was 78.28 N; and for bending modulus and bending strength were 58.5 MPa and 3510 MPa respectively (Figure 5B).

In group A, after 4 weeks, 34.8% of the entire defective area was filled with new bony tissue confirmed histologically. In group B, 30% of the defects were replaced with new bony tissue and in the control group 10% were filled with new bony tissue. The new bony tissue formed in group A was more than group B but the difference was not statistically significant. In both groups, the difference was significant in comparison to the control group (p=0.001).

**Fig. 6:**
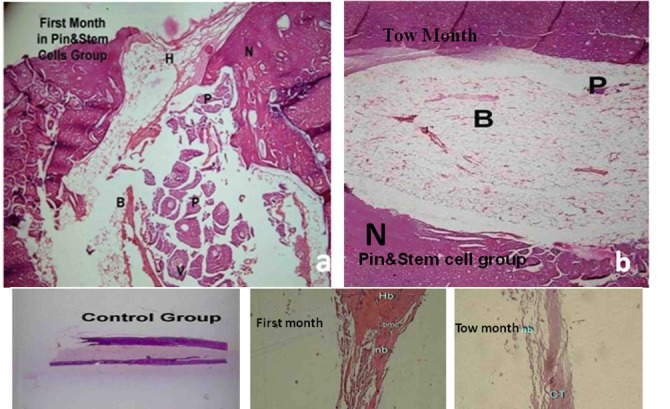
Photomicrographs of H and E stained histological sections from bone defect. A. New bone tissue and few vascular cavities were formed adjacent to the pin edge after 4 weeks post-implantation. B. After 8 weeks, defects grafted with pin and pin together with MSCs could almost be completely filled with new bony tissue. Bony tissue formed in internal side of the defect was completely remodeled. A new bone was present within the marrow cavity that was initially filled with bony tissue (x40) C. Gross appearance of the control group. D, E. The control group after 4 and 8 weeks post-implantation which was primarily filled with less connective tissue and showed the minimal new bone formation (x10). H: The fracture site, N (nb): New bone, P: Pin, B: Bone marrow, V: vascular cavity, CT: connective tissue, Hb: host bone.

**Fig. 7:**
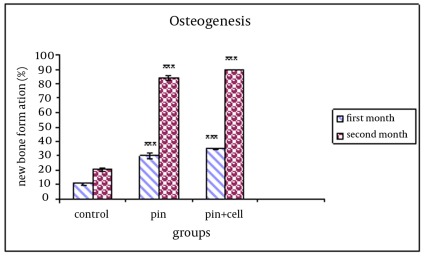
Comparison of the measure of new bone formation quantified by histomorphometric analysis in groups which showed evidence of osteogenesis following 4 and 8 weeks post implantation as compared to control group.

In group A after 8 weeks, 89.5% of the defects were filled with new bony tissue. This figure for group B and the control group was 83.9% and 20% respectively. The difference was statistical significant between groups A and B and between these groups and the control group (p=0.001). Table 1 and Figure 6 and Figure 7 illustrate the histological findings of new bony tissue formed in the three groups.

**Table 1:** Osteogenesis after use of pin and pin together with MSCs after 4-8 weeks post-implantation in comparison to the control group using histological analysis (mean±SEM; n=5 in each group).

**Osteogenesis (New bone formation %)**		
**Experimental group**	**After 4 weeks**	**After 8 weeks**
Pin	30.06±0.9357	83.96±1.450
Pin and MSCs	34.86±2.002	89.56±1.481
Control	10.49±0.3731	20.19±0.5324

**Fig. 8:**
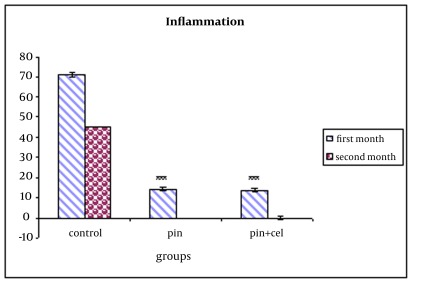
Comparison of inflammation quantified by histomorphometric analysis in groups which showed evidence of a decrease in inflammation following 4 and 8 weeks post implantation as compared to control group. The error bar indicate SE for n=5, p value <0.001 are indicated by ***.

After 4 weeks, inflammation was just visible in 13.9% of group A and 14.3% of group B in comparison to 71% of the control group showing a healing trend in groups A and B but the difference between groups A and B was not statistically significant (p>0.05) (Figure 7). The difference was statistically significant between groups A and B and the control group (p=0.001). After 8 weeks, there were no inflammation visible in the both A and B groups in comparison to the control group (p=0.001) (Table 2, Figure 8) showing a healing trend in groups A and B. The scaffold bending test for the bending modulus and strength was as follows: d=1 mm, h=1 mm, b=1 mm, L=30 mm, W=30, F=78 N, δ=3FL/2bd2= 3510Mpa and e=L3F/4wh3d=58.5MPa

**Table 2:** Degree of inflammation in pin and pin together with MSCs after 4-8 weeks post-implantation in comparison to the control group using histological analysis (mean±SEM; n=5 in each group)

**Degree of inflammation (%)**		
**Experimental group**	**After 4 weeks**	**After 8 weeks**
Pin	14.38±0.7921	0
Pin and MSCs	13.94±0.7514	0
control	71.2±0. 9638	45.5±0.2587

## Discussion

The repair of bone defects by using stem cells is still a major challenge in tissue engineering.[16][17][18] Bone marrow-derived MSCs promote bone repair when implanted locally, usually on a scaffold such as hydroxyapatite/tricalcium phosphate or hydroxyapatite ceramic. In the case of long bone defects, a porous ceramic scaffold together with MSCs was shown to complete unification between implant and bone after a 5-6 months period.[9][13][19] Peterson et al. also showed that a collagen-ceramic carrier seeded with MSCs derived from human adipose tissue could be implanted into large femoral bone defects of rats and after 8 weeks could promote the healing.[20] The scaffold used in this study was originated from human femoral bone which was used together with MSCs and their ability in the regeneration of bony tissue was evaluated. The results revealed that MSCs played a crucial role in repair of bone defects. Our data demonstrated that new bone formation and step of maturity were significantly more, when the scaffold was used with MSCs. Considering inflammation, better results were observed in groups using pin alone and pin together with MSCs in comparison to the control group, but no statistical difference was noticed between unloaded scaffolds and those scaffolds loaded with MSCs. The histological evaluation to determine the biodegradation of the scaffolds showed a faster resorption of pin in both groups using pin alone and pin together with MSCs after 8 weeks.

It was shown that bone tissue derived from human MSCs was at the center of transplants, but not at the periphery.[21] Bone tissue at the periphery and the bone marrow were identified as a host origin.[21] However, in our study in both groups, new bone formation was noticed in the periphery and center of transplants, but in the control group, less new bone formation was detected at the periphery. These data suggest that new bone formation is limited by the availability of recipient-derived bone forming cells. Rudimentary results of this study regarding a better healing rate in bone defects appear promising. Investigation of the regenerative potential of transplanted pin together with MSCs in animal model was a first step in therapeutic application of this pin together with MSCs in the clinical practice. Therefore, tissue engineering of mineralized bone xenograft and MSCs allograft may be significant steps in bone healing and regenerative medicine.
